# Editorial: Antimicrobial resistance and therapy in critically ill patients, volume II

**DOI:** 10.3389/fmed.2026.1889971

**Published:** 2026-06-25

**Authors:** Xiao-Hong Zhou, Zhi-Tao Yang, Shun-Jin Zhao, Qi Li, Jian-Cang Zhou

**Affiliations:** 1 Department of Ultrasound, Lanxi People's Hospital, Lanxi, China; 2 Department of Emergency Medicine, Ruijin Hospital, Shanghai, China; 3 Department of Respiratory and Critical Care Medicine, Lanxi People's Hospital, Lanxi, China; 4 Department of Emergency Medicine, Lanxi People's Hospital, Lanxi, China; 5 Department of Critical Care Medicine, Sir Run Run Shaw Hospital, Zhejiang University School of Medicine, Hangzhou, China

**Keywords:** antimicrobial resistance, carbapenem-resistant organisms, critically ill, therapeutic drug monitoring, therapy

Multidrug-resistant (MDR) pathogens infections have increasing been a pressing challenges in critically ill patients. Antimicrobial resistance, limited therapeutic options, and heterogeneous pharmacokinetic responses in unstable patients continue to drive excess mortality and treatment failure (1). This special topic brings 13 high-quality original investigations, clinical studies, and trial protocols focused on critical care infections, spanning antimicrobial pharmacology, precision dosing, and novel disinfection strategies. Collectively, these works strengthen the evidence for personalized, mechanism-driven care and offer actionable solutions to improve outcomes for the critically ill patients.

In this Research Topic, four articles explored the optimal use of last-line antibiotics to combat MDR Gram-negative bacteria (MDR-GNB), a major cause of life-threatening nosocomial infection (2). First of all, Liu H. et al. provided a head-to-head comparative data for colistin sulfate vs. polymyxin B in carbapenem-resistant *Acinetobacter baumannii* infections, reporting comparable 28-day mortality (32.8% vs. 37.8%) but significantly lower risk of acute kidney injury with colistin sulfate (5.2% vs. 19.5%), highlighting a safer alternative for patients at high renal risk. Zhuang et al. presented a multicenter real-world study evaluating polymyxin B for stroke-associated pneumonia caused by MDR-GNB, demonstrating that adjunctive aerosolized administration significantly improved microbial eradication compared with intravenous therapy alone (46.7% vs. 26.7%). This finding supported inhaled polymyxins as a valuable adjuvant strategy for severe pneumonia in vulnerable populations. In a meta-analysis included a total of 74 studies and 8,889 participants, Li et al. demonstrated that the loading dose, combined therapy with carbapenems or quinolones, microbial factors, and baseline health status are the main factors associated with the effectiveness of colistin. Additionally, all these factors are associated with an increased risk of colistin-associated AKI.

Within this Research Topic, two clinical trial protocols were also included. Liu J. J. et al. introduced a rigorously designed multicenter randomized controlled trial protocol comparing efficacy and safety of eravacycline and tigecycline for complicated intra-abdominal infections in the ICU, addressing a critical evidence gap for patients with multidrug-resistant pathogens. While Momčilović et al. detailed an ongoing randomized trial comparing continuous infusion vs. intermittent dosing of ceftazidime/avibactam for infections caused by OXA-48 producing *Klebsiella pneumoniae* or *Pseudomonas aeruginosa*, exploring whether prolonged infusion improves PK/PD target attainment and microbiological success.

Although pharmacokinetics of antimicrobial agents are altered in critically ill patients, precision therapeutic drug monitoring has also advanced substantially in this topic (3). Dreyse et al. conducted a prospective cohort study validating Bayesian AUC-guided vancomycin dosing in critically ill patients, showing that AUC estimates incorporated peak and trough plasma concentrations demonstrated significantly greater accuracy and lower bias than single trough-level monitoring. In a retrospectively study which included 270 patients, Hellwege et al. found that daptomycin levels might be commonly low (54.4%) in critically ill patients and often appear not to increase after dose adjustments. Patients who underwent therapeutic drug monitoring (TDM) experienced significantly more frequent daptomycin dose increases than those who did not (28.8% vs. 13.1%, *p* = 0.002). Voriconazole (VCZ) was primarily metabolized by cytochromeP450 (CYP450) 2C19, with secondary pathways including CYP3A4 and CYP2C9, this meaning that any factors that may affect liver enzyme activity could be related to its serum concentration. Chen et al. demonstrated CRP is a significant positive predictor of VCZ trough concentration. Although loading dose was correlated with increased trough concentration, it was also elevated the risk of supratherapeutic levels. These works align with updated international guidelines and promote safer, more target-attainment dosing for severe Gram-positive infections. Therefore, these pharmacokinetic and pharmacodynamic insights shift the field toward truly individualized antibiotic dosing rather than standardized weight-based regimens (4).

Pneumonia is a very common complication for many diseases such as stroke and traumatic brain injury. Amati et al. performed a propensity score-matched analysis and demonstrated that postoperative pulmonary complications after emergency laparotomy were common and associated with significantly higher 90-day mortality. Independent preoperative predictors included higher ASA score, elevated C-reactive protein-to-albumin ratio, lower hemoglobin level, and multidrug-resistant organism (MDRO) colonization. Lin et al. conducted a retrospective observational cohort study and found that high lateral position was independently associated with shorter antibiotic exposure duration in patients with severe traumatic brain injury complicated by aspiration pneumonia. High lateral position also showed favorable trends toward fewer mechanical ventilation days and a lower tracheostomy rate, with acceptable safety and feasibility in clinical practice.

Some opportunistic and rare pathogens were also explored in the Research Topic. Shi et al. reported a prospective cohort describing the clinical course, treatment responses, and outcomes of severe pneumocystis pneumonia in patients with autoimmune and inflammatory diseases, identifying early treatment failure, progressive pulmonary fibrosis, and severe hypoxemia as strong predictors of 28-day mortality. Huang et al. reported a case of severe pneumonia caused by *Cupriavidus gilardii* in a critically ill, immunocompromised 75-year-old male patient. The pathogen was difficult to identify and lacked standardized antimicrobial susceptibility testing, leading to initial ineffective empirical therapy. Targeted treatment with cefoperazone–sulbactam plus minocycline achieved clinical improvement, highlighting the need for standardized guidelines and multidisciplinary care for rare environmental pathogens.

Beyond antibiotics, innovative non-antimicrobial infection control approaches emerged as a transformative theme. Wang et al. demonstrated that cold atmospheric-pressure plasma effectively inactivates four major clinically relevant multidrug-resistant pathogens—including carbapenem-resistant *Acinetobacter baumannii*, carbapenem-resistant *Pseudomonas aeruginosa*, methicillin-resistant *Staphylococcus aureus* (MRSA), and carbapenem-resistant *Klebsiella pneumoniae*—on hospital surfaces and within simulated ward environments. Mechanistically, cold atmospheric-pressure plasma (CAP) acts by disrupting bacterial membrane integrity and intracellular structures, offering a safe, sustainable, no-touch environmental disinfection strategy, which is a promising technology for reducing cross-transmission in high-risk ICU settings (5).
